# Glitazone use associated with reduced risk of Parkinson's disease

**DOI:** 10.1002/mds.27128

**Published:** 2017-09-01

**Authors:** Brage Brakedal, Irene Flønes, Simone F. Reiter, Øivind Torkildsen, Christian Dölle, Jörg Assmus, Kristoffer Haugarvoll, Charalampos Tzoulis

**Affiliations:** ^1^ Department of Neurology Haukeland University Hospital Bergen Norway; ^2^ Department of Clinical Medicine University of Bergen Bergen Norway; ^3^ Centre for Clinical Research Haukeland University Hospital Bergen Norway

**Keywords:** thiazolidinediones, neuroprotection, mitochondria, pioglitazone, rosiglitazone, neurodegeneration, peroxisome proliferator‐activated receptor gamma (PPARγ)

## Abstract

**Background:**

Whether antidiabetic glitazone drugs protect against Parkinson's disease remains controversial. Although a single clinical trial showed no evidence of disease modulation, retrospective studies suggest that a disease‐preventing effect may be plausible. The objective of this study was to examine if the use of glitazone drugs is associated with a lower incidence of PD among diabetic patients.

**Methods:**

We compared the incidence of PD between individuals with diabetes who used glitazones, with or without metformin, and individuals using only metformin in the Norwegian Prescription Database. This database contains all prescription drugs dispensed for the entire Norwegian population. We identified 94,349 metformin users and 8396 glitazone users during a 10‐year period and compared the incidence of PD in the 2 groups using Cox regression survival analysis, with glitazone exposure as a time‐dependent covariate.

**Results:**

Glitazone use was associated with a significantly lower incidence of PD compared with metformin‐only use (hazard ratio, 0.72; 95% confidence interval, 0.55‐0.94; *P* = 0.01).

**Conclusions:**

The use of glitazones is associated with a decreased risk of incident PD in populations with diabetes. Further studies are warranted to confirm and understand the role of glitazones in neurodegeneration. © 2017 The Authors. Movement Disorders published by Wiley Periodicals, Inc. on behalf of International Parkinson and Movement Disorder Society

Parkinson's disease (PD) is one of the most common neurodegenerative disorders and a major cause of death and disability. PD affects ∼2% of the population older than 65 years, and its prevalence is increasing as the population ages.^1^, ^2^ The etiology of PD is unknown, and despite good symptomatic treatments, there is no cure and patients die prematurely because of progressive disability.

Substances activating peroxisome proliferation‐activated receptor gamma (PPARγ) have shown neuroprotective properties in both in vitro and in vivo models of neurodegeneration. In particular, the antidiabetic drugs thiazolidinediones, also known as glitazones (GTZ), have been shown to rescue dopaminergic neuronal loss, decrease neuroinflammation, and ameliorate motor phenotypes in MPTP or rotenone‐based animal models of parkinsonism.^3^, ^4^, ^5^, ^6^, ^7^ Toxin‐based models do not accurately reflect PD pathogenesis, however, and the results from these experiments are difficult to interpret in the context of human disease.

In a recent randomized, controlled trial, pioglitazone showed no significant effect on disease progression in 210 patients with early PD over a period of 44 weeks.^8^ Two cohort studies have been conducted with apparently contradictory results. A retrospective study in a large UK cohort of patients with diabetes found that chronic GTZ use was associated with a reduction in the incidence of PD by about 28%,^9^ whereas another study in a North American cohort found no significant risk modification.^10^

Based on current evidence, it remains unclear whether GTZs have a neuroprotective effect in PD. To address this question, we performed a long‐term retrospective cohort study using GTZ drugs assessing the incidence of PD among people with diabetes mellitus from the entire Norwegian population.

## Methods

### Ethical Considerations

The study was approved by the Regional Committee for Medical and Health Research Ethics, Western Norway (REK 2014/2164).

### Materials

Our study was based on the Norwegian Prescription Database (NorPD), an unselected, population‐based registry of all drug prescriptions dispensed from Norwegian pharmacies to individual patients. Drugs dispensed in institutions are not included.^11^ The NorPD comprises a complete record of every dispensing of prescribed medication from pharmacies since January 1, 2004, for the entire Norwegian population (5.1 million in 2013). The clinical indication for each prescription is registered in the form of either a diagnosis code from the International Classification of Diseases, 10th revision, and/or the International Classification of Primary Care, 2nd edition, or a disease‐ or disease‐group‐specific reimbursement code. Medication for PD and diabetes is always reimbursed in Norway and strictly prescription controlled. Therefore, all patients receiving treatment with a PD diagnosis are included. A unique personal identifier enables consecutive monitoring of individuals in the health system over the entire life span. A complete record of deaths is included in the NorPD database.^11^

### Study Design and Selection of Groups

We hypothesized that use of GTZs would be associated with a lower incidence of PD in our population. To avoid the confounding effect of diabetes, we studied only diabetic patients who used GTZ or metformin in the period January 1, 2005, to December 31, 2014.

**Figure 1:**
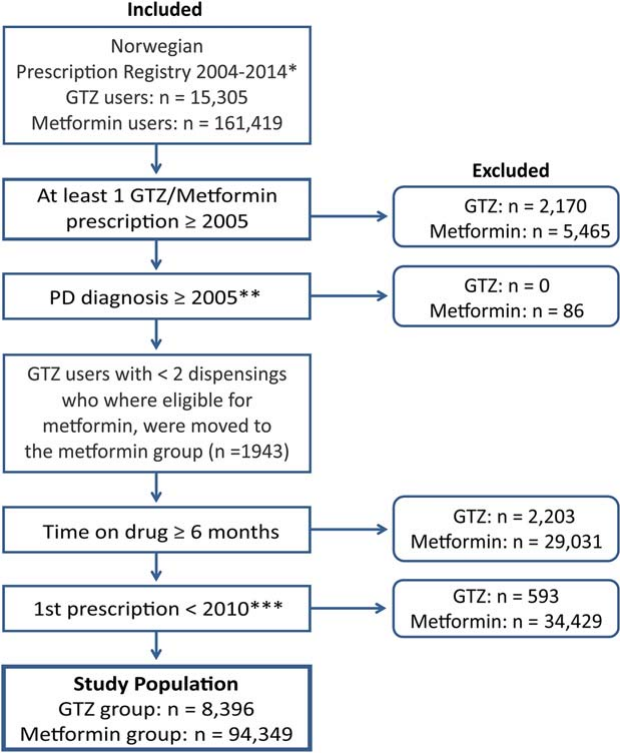
Study population. Flow chart showing definition of the study population and reasons for exclusion. *Total number of individuals who, in the period January 1, 2004, to December 31, 2014, received at least 1 prescription of GTZ or metformin.**From Januar 1, 2005. ***Before January 1, 2010. [Color figure can be viewed at wileyonlinelibrary.com]

GTZ and metformin users were defined based on receiving prescriptions of the respective drugs. The codes used to identify prescriptions of GTZ and metformin are summarized in the Supplementary Information. To be classified as GTZ or metformin users, individuals had to be dispensed GTZ or metformin, respectively, at least 2 times consecutively. The interval between consecutively dispensed medications was defined as at least 1 month (30 days) and less than 6 months (180 days). All individuals had to use GTZ or metformin for more than 6 months to be included as GTZ or metformin users, respectively. To ensure incident use of GTZ, we only included users who received their first dispensing of GTZ drugs at least 12 months after baseline (January 1, 2004). Because GTZ drugs are a second‐line treatment for type II diabetes mellitus, individuals could be using metformin (a first‐line treatment) from baseline, and these were eligible GTZ users. Once individuals were classified as GTZ users, they stayed in that group for the remaining follow‐up period. To differentiate between ongoing and previous GTZ exposure among GTZ users, their follow‐up period was subdivided into current GTZ and past GTZ exposure time. A 3‐month washout period after the last GTZ prescription was included when shifting from current to past GTZ exposure status. Individuals in the metformin group were not exposed to GTZ. The age requirement for the groups was set between 40 and 100 years. Inclusion criteria are summarized in Figure 1.

The outcome (end points) of the study was the first entry of either the end date of the study (December 31, 2014), time of death, or incident PD. The follow‐up time for the GTZ users was defined from the index date of the study to an outcome (end point).

**Figure 2:**
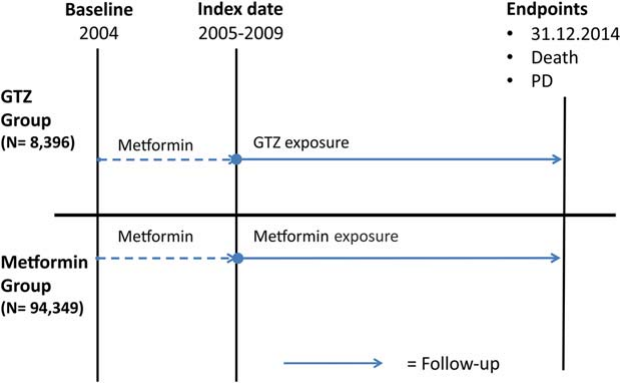
Graphical presentation of follow‐up time. Baseline (ie, earliest data registration date in NorPD) is January 1, 2004. Individuals who received a GTZ or PD drug during 2004 were excluded to ensure incident use. The stippled line indicates that individuals could be using metformin in 2004. Index date (ie, the start of follow‐up) for GTZ and metformin users is the date of the first drug dispensing after inclusion to the GTZ or metformin group, respectively. Follow‐up time is from the index date to 1 of 3 possible end points: end of study observation (December 31, 2014), death, or incidence of PD. [Color figure can be viewed at wileyonlinelibrary.com]

The index date of GTZ or metformin exposure was set to the first dispensed prescription of GTZ or metformin, respectively, after December 31, 2004. The first occurrence of an end point was registered after a subject had been classified as a GTZ or metformin user. Duration of GTZ and metformin exposure was defined as the cumulative time (in months) an individual was exposed to GTZ or metformin, respectively, from the index date. Rosiglitazone was suspended in 2010 in Norway because of cardiovascular safety concerns. This had a substantial impact on prescription practices of GTZ drugs. Inclusion of metformin and GTZ users was therefore set until December 31, 2009. The total time an individual used GTZ or metformin was calculated from the cumulative time between consecutive prescriptions. In individuals who had interrupted and restarted their treatment, the total exposure time of all individual periods of consecutive prescriptions was calculated. The design of the study is summarized in Figure 2.

### Identification of Patients With PD

Incident PD was defined as the time of the first of at least 2 consecutive prescriptions of a PD medication, given with a registered diagnosis of PD (Supplementary Information). All medication for PD is reimbursed in Norway when coupled and registered with a PD diagnosis. To ensure incident cases of PD, we excluded those individuals who received PD medications the first 12 months after baseline (January 1, 2004, to December 31, 2004). Patients with a PD diagnosis prior to either a first GTZ or metformin prescription were excluded from the study.

### Statistical Methods

We compared the incidence of PD among individuals with diabetes during and after GTZ exposure to metformin only over a 10‐year period (January 1, 2005, to December 31, 2014). We applied Kaplan‐Meier estimators to study the PD‐free survivor functions dependent on GTZ exposure. The Kaplan‐Meier survivor function measures the incidence of PD in the GTZ and metformin groups from the start of GTZ and metformin exposure, respectively. The Breslow test was used to test equality of the survivor functions. The GTZ users' follow‐up time was grouped into current and past GTZ exposure time, and additional Kaplan‐Meier survivor estimators were performed to study the PD‐free survivor functions during current and past GTZ exposure compared with metformin. We performed a time‐dependent Cox regression analysis where exposure to GTZ was time dependent to eliminate immortal time bias among GTZ users (follow‐up time from metformin use to GTZ exposure). The inclusion in the GTZ group was defined from the onset of GTZ exposure. The model was adjusted using the fixed covariates age and sex. The event times were censored at the time of death. We estimated the hazard ratio (HR) and 95% confidence interval (CI) for the incidence of PD between GTZ and metformin users. The age and sex covariates had a significant contribution to the model. The covariates (age and sex) satisfied the proportional hazard assumption, which was verified by examining the DFBETA residuals against time and log(‐log[PD incident]) versus log(time) curves. All data processing and analyses were performed using IBM SPSS Statistics version 23.0 (SPSS Inc., Chicago, IL).

## Results

### Descriptive Results

There were 15,305 users of GTZ and 161,419 users of metformin in the period 2004‐2014. Of these, 8,396 GTZ users and 94,349 metformin users fulfilled the inclusion criteria (Fig. 1). The GTZ users had a cumulative follow‐up time of 69,338 patient‐years, of which 29,645 were during current GTZ exposure. Metformin users had a total follow‐up time of 657,537 patient‐years. During follow‐up time, there were 57 and 938 instances of incident PD among the GTZ and metformin users, respectively. In total, 3,864 GTZ users were exposed to GTZ for ≤36 months. Among them were 34 incident cases of PD. Of these, 13 occurred during the GTZ users' current GTZ exposure and 21 after GTZ discontinuation. In total, 4,532 GTZ users were exposed to GTZ for >36 months. Among them were 23 incident cases of PD. Of these, 4 occurred during current GTZ exposure and 19 after GTZ discontinuation. There were no incident cases of PD among current GTZ users who had been exposed to GTZ for >46 months (n = 2,935 individuals, 5,557 patient‐years). Among the GTZ users, 7576 individuals (90.2%) used metformin before GTZ. In total, 8,020 GTZ users (95.5%) used metformin sometime during the follow‐up period. Patient demographics and descriptive statistics are summarized in Table 1.

**Table 1:** Patient demographics and descriptive statistics for database

Demographics and descriptive statistics	Glitazones	Metformin only
Number of individuals	8,396	94,349
Follow‐up (patient‐years)	69,338	657,537
Age, mean (SD)	62.6 (10,7)	64.3 (11.6)
Deaths (%)	1,494 (17.7%)	23,241 (24.6%)
Parkinson's disease, n (%)	57 (0.7%)	938 (1.0%)
Sex (%)		
Female	3,464 (41.2%)	42,778 (45.3%)
Male	4,932 (58.8%)	51,571 (54.7%)
Users of metformin, n (%)	8,020 (95.5%)	94,349 (100%)
Users of metformin before GTZ, n (%)	7,576 (90.2%)	
Users by duration of GTZ use, n (%)		
<2 Years	2,620 (31.2%)	
2‐4 Years	2,970 (35.4%)	
>4 Years	2,806 (33.4%)	
Duration of drug use (months), mean (SD)		
Metformin	85.3 (40,0)	78.3 (36.0)
GTZ	39.4 (24.0)	
Follow‐up time (months), mean (SD)	83.3 (24.3)	83.6 (29.7)
Users of GTZ (%)		
Pioglitazone	1,303 (15.5%)	
Rosiglitazone	6,278 (74.8%)	
Pioglitazone and rosiglitazone	815 (9.7%)	
Users of metformin in 2004 (%)	5,526 (65.8%)	42,903 (45.5%)
Calendar year at index date (%)		
2005	2,621 (31.2%)	52,669 (55.9%)
2006	2,601 (31.0%)	10,425 (11.0%)
2007	1,509 (18.0%)	10,570 (11.2%)
2008	914 (10.9%)	10,513 (11.1%)
2009	751 (8.9%)	10,172 (10.8%)
Final year of drug (GTZ/metformin), n (%)		
2005‐2007	1,393 (16.6%)	10,148 (10.7%)
2008‐2009	2,022 (24.0%)	9,663 (10.2%)
2010	3,741 (44.6%)	6,694 (7.9%)
2011‐2014	1,240 (14.8%)	68,144 (72.2%)

GTZ, glitazones; SD, standard deviation; PD, Parkinson's disease.

Final year of drug is the year of the last prescription of a drug (GTZ/metformin).

### Statistical Analysis

**Figure 3:**
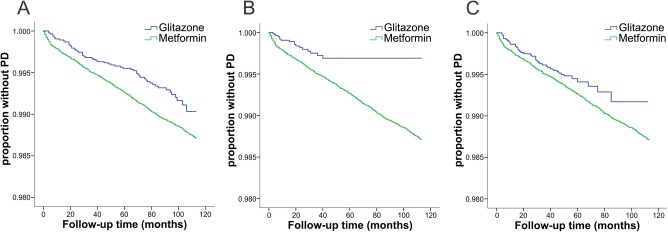
Kaplan‐Meier survival curves. (A‐C) Kaplan‐Meier estimators of time to PD diagnosis as a function of treatment duration (in months) with GTZ (blue) and metformin (green). (A) GTZ users (current and past), (B) current GTZ exposure, (C) past GTZ users.

Kaplan‐Meier analysis showed a significantly lower incidence of PD in the GTZ users compared with metformin users (*P* = 0.003; Fig. 3A). The time‐dependent Cox regression model showed that GTZ exposure was associated with a 28% reduction in risk of incident PD compared with metformin exposure (HR, 0.72; 95% CI, 0.54‐0.94; *P* = 0.015). Overall, males had a 48% higher risk than females of developing PD (HR, 1.48; 95% CI, 1.30‐1.68). The results of the Cox analysis are shown in Supplementary Table 1.

The Kaplan‐Meier analysis showed that current GTZ exposure was associated with a significantly lower incidence of PD compared with metformin‐only exposure (*P* = 0.001), whereas no difference was found for past exposure (*P* = 0.10); see Figure 3B,C.

## Discussion

We performed a long‐term retrospective cohort study investigating the association between incident PD and GTZ use in an unselected sample representing the entire Norwegian population. Our findings show that GTZ use was associated with a 28% decrease in the risk of developing PD compared with metformin use in a nationwide population using these drugs for diabetes mellitus. Moreover, we observed that there was a lower incidence of PD during current GTZ exposure compared with past GTZ exposure.

The results of other human studies on GTZ and PD have been conflicting. One retrospective study, conducted on a subset of the UK population, showed a significantly reduced risk for PD among GTZ users, with an overall effect of similar size (28% risk reduction) to our population.^9^ Interestingly, Brauer et al also suggested an association between lower incidence of PD and current GTZ use. In contrast, no significant association was found between GTZ use and the incidence of PD in a US Medicare population.^10^ However, this study was based on a selected sample from an insurance claims database and cannot be regarded as representative of the general population. Moreover, it may have been underpowered for detecting a protective GTZ effect because of a smaller sample and shorter mean observation time (2.97 years) compared with our study (6.94 years) and the study by Brauer et al (6.1 years).

A phase 2 randomized, double‐blind clinical trial assessing the effect of pioglitazone on the progression of newly diagnosed PD over a period of 44 weeks (ClinicalTrials.gov, registration: NCT01280123) concluded that pioglitazone was unlikely to modify disease progression in early PD.^8^ However, that study was not designed to assess the reduction in incidence of PD among GTZ users. Moreover, because of a short duration of pioglitazone use and follow‐up period, the study would not detect any long‐term neuroprotective or disease‐modifying effects. Our observation that there was no incidence of PD among current GTZ users who had been exposed to GTZ for >46 months (Fig. 3B) may be in line with this.

It is possible that the neuroprotective action of GTZ requires long‐term exposure, starting at preclinical stages of the disease, before neurodegeneration has advanced sufficiently to cause clinical dysfunction. From a biological standpoint, this would be a plausible hypothesis, as PD has a long prodromal phase, and the majority of neuronal populations affected by the disease have already degenerated at clinical onset.

As our entire cohort had diabetes, it is not possible to generalize our findings for the general population. However, whether diabetes influences the risk of developing PD remains controversial. One meta‐analysis of cohort studies has found a positive association, whereas another for PD meta‐analysis of case‐control studies suggested patients with diabetes may have a decreased incidence of PD.^12^, ^13^ Interestingly, the calculated annual incidence rate for PD in our metformin group was 14.2 per 100,000 patient‐years, which is very similar to that reported in the general Norwegian population (12.6/100,000).^14^ However, the incidence rate of PD in our GTZ group was much lower, at 8.2 per 100,000 patient‐years.

The mechanisms underlying the possible neuroprotective effect of GTZ are currently undetermined. One possibility is that GTZ acts by improving mitochondrial function. GTZ drugs stimulate the PPARγ, which, via the PPARγ coactivator 1‐α pathway, leads to increased mitochondrial biogenesis.^15^ This could partly compensate for the mitochondrial pathology that characterizes PD such as accumulation of somatic mitochondrial DNA (mtDNA) damage^16^ and respiratory deficiencies.^17^ It is possible, that GTZ drugs ameliorate these defects by increasing mtDNA synthesis and overall mitochondrial mass. GTZ may also have an anti‐inflammatory action via decreased microglial activation and inhibition of tumor necrosis factor α as well as reduced nitric oxide‐mediated toxicity.^4^, ^7^

Irrespective of the mechanisms involved, our results suggest that GTZ use is associated with lower incidence of PD, but it may require long‐term and current exposure to GTZ. Our study was designed to detect an association with the incidence of PD and cannot predict whether GTZ exposure has a disease‐modifying effect after the onset of clinical disease. Further studies are required to assess whether GTZ truly has a protective effect against PD and to understand the underlying mechanisms so that these may be exploited therapeutically.

### Strengths and Limitations

The primary strength of our study is that the Norwegian Prescription Database (NorPD) includes all GTZ prescriptions dispensed from pharmacies to individual patients within Norway and is coupled with a complete record of all deaths. We therefore have a long and representative follow‐up time for all GTZ users. A potential limitation to this study is that we have used a by‐proxy definition of PD. To reduce this possible confounder, we used a strict definition of PD, in which subjects had to receive at least 2 prescription of a PD medication coupled with a PD diagnosis (reimbursement code). Earlier studies had found a similar definition to have a high positive predictive value for PD (91%).^18^ Moreover, our by‐proxy defined PD population has a male‐to‐female ratio similar to that described in the general population, further validating our method.^14^, ^19^ In the early stages of parkinsonism, it may be difficult to differentiate between PD and secondary causes of parkinsonism as well as atypical parkinsonism syndromes.^20^ However, the incidence of vascular parkinsonism and atypical parkinsonism syndromes is much rarer than PD, and we do not believe it would significantly bias our results.^14^, ^21^

Our results remained significant after correcting for age and sex. Lack of information regarding treatment stage of diabetes is a potential limitation in our study. However, as diabetes has not been shown to have a definite effect on the risk for PD, we find it unlikely that treatment stage would significantly bias our results. A potential effect of further confounders cannot be excluded in our study, as this information is not included in the NorPD database. Multiple additional covariates were included in the 2 previous studies, but none were found to have significant impact. Therefore, it is unlikely that the addition of more covariates would change our overall findings.^9^, ^10^ Rosiglitazone was suspended in Norway in 2010 because of suspected risk for cardiovascular adverse events. This changed the prescription practices of GTZ from 2010. We therefore did not include new GTZ users after 2010.

Finally, the dose‐effect relationship needs to be explored. In our study, we could not correct for the dosage of GTZ or metformin medication, as these data were not available to us. It remains unknown whether the observed effect of GTZ therapy is dosage dependent.

## Author Contributions

B.B. — conception, design, and execution of statistical analysis; writing of the first draft and review and critique of the manuscript.

I.F. — design and execution of statistical analysis; review and critique of the manuscript.

S.F.R. — design and execution of the statistical analysis; review and critique of the manuscript.

Ø.T. — design of the statistical analysis; review and critique of the manuscript.

C.D. — organization and design of the research project; design of the statistical analysis; review and critique of the manuscript.

J.A. —organization of the research project; design of the statistical analysis; review and critique of the manuscript.

K.H.— organization and execution of the research project; review and critique of the manuscript.

C.T. —conception, organization, funding, and execution of the research project; writing of the first draft and review and critique of the manuscript.

## Competing interests

The authors declare that they have no competing interests

## Full financial disclosure for the previous 12 months

C.T. receives research support from the Western Norway Regional Health Authority and the Research Council of Norway. K.H. receives research support from the Research Council of Norway and the Dystonia Medical Research Foundation. I.F. and C.D. receive research support from the Western Norway Regional Health Authority. S.F.R. receives research support from the University of Bergen.

## Supporting information

Additional Supporting Information may be found in the online version of this article at the publisher's website.

Supporting Information Table 1.
