# Supplementary material for: Glitazone use associated with reduced risk of Parkinson's disease
Source: Mov Disord. 2017 Sep 1;32(11):1594–9. doi: 10.1002/mds.27128 (PMC5697685; doi:10.1002/mds.27128)
Supplement: Supplementary file 1 — Supporting Information Table 1. [file MDS-32-1594-s001.docx]

**Supplementary Table**

**Supplementary Table 1. Primary analysis: Cox regression and calculated Hazard Ratios (HR)**

|  | B | P value | HR | 95% CI for HR | |
| --- | --- | --- | --- | --- | --- |
| GTZ/metformin* | -0.33 | 0.015 | 0.72 | 0.55 | 0.94 |
| Male/female** | 0.39 | <0.001 | 1.48 | 1.30 | 1.68 |
| Age | 0,04 | <0.001 | 1.04 | 1.04 | 1.05 |

*GTZ group compared to metformin group, calculated as time-dependent variable. ** Males compared to females. HR: hazard ratio. CI: 95% confidence interval.
